# Research and Remedies

**Published:** 1912-06-01

**Authors:** 


### RESEARCH AND REMEDIES.

### The "Antiseptic" (?) Shaving-block.

The dangers to their customers which attend the use of the "alum block" by barbers are emphasised in an article from the pen of Dr. Remlinger in a recent number of the *Revue d'Hygiène*. He took possession of one of these blocks which had been in use for two months in a shaving saloon, and soaked it for five minutes in 50 c.c. of distilled water, afterwards repeating the immersion in another bath. The block yielded 68,250 colonies of aerobic bacteria to the first, and 59,150 to the second bath, and among the bacteria recognised were the staphylococcus, the bacillus mesentericus, a liquefying fluorescent bacillus, yeasts, and various fungi. On experimenting with a new unused block he succeeded in growing cultures upon it of tetanus, tubercle, staphylococcus, Loeffler's bacillus, anthrax, glanders, etc., which upon subsequent inoculation into guinea-pigs produced a fatal ending. Now, these "blocks" have so far enjoyed a reputation among barbers of being antiseptic by virtue of the small amount of boric acid mixed with the glycerine and alum of which they are composed. The author's researches have completely upset this supposition. He advises that instead of employing a common block for all customers, small pastilles of alum should be used, a fresh one for each customer.

### Sodium Salicylate as an Antiseptic.

Sapojkov has recently drawn attention to the efficacy of sodium salicylate as an antiseptic dressing in cases of furunculosis and anthrax. To obtain the full benefit of the drug it is necessary that the affected part be denuded of epithelium, if this is not already the case. To that end he gently scarifies the skin round the furuncle and then applies the drug freely in a finely powdered condition to the denuded area. Within twenty-four hours excellent results are obtained, pus being freely discharged from the furuncle, and only slight pressure is necessary to cause the exit of the comedo from the deeper tissues. The author considers this method superior to that of Bier because of its simplicity and because it can be applied to all parts of the body. The only drawback to its use lies in the fact that a sharp burning pain is experienced when it is applied to the denuded surface. He has made use of the drug as an insufflation in cases of tonsillitis, but reports that its disagreeable taste often induces patients to refuse to undergo a systematic course of treatment. When patients can be induced to persevere the results in such cases are excellent.

### The Prognosis of Chronic Bright's Disease.

In the *Journal of Clinical Research* attention is directed to a method of estimating the imminence, or otherwise, of the final catastrophe in cases of chronic Bright's disease, whether tubal or interstitial. This has been devised by Widal, and depends on an analysis of the quantity of urea present in the circulating blood. A sample is taken from a peripheral vein in the same way as for a blood culture, and the subsequent proceedings can be conducted in a clinical laboratory. The limits of normal variation are from 0.15 to 0.5 gramme of urea per litre of serum. Anything above the latter figure indicates a condition which requires a most guarded prognosis; with anything above 1.0 gramme per litre the prospect is gloomy, and over 2.0 means certain death at no very distant date. Of thirty patients whose urea content exceeded this latter figure, twelve died within a week, and eight more within a month; all the remainder died before the eighth month. When the urea is found to be below 0.5 gramme per litre, the prognosis is relatively good; this figure may even be temporarily exceeded, up to 1.0 gramme without any serious complication necessarily following, but the tendency is for the quantity to rise as time goes on, and the outlook becomes correspondingly less hopeful.

